# Evaluation of accuracy, reliability, quality, and readability of online patient information materials on coccyx injury

**DOI:** 10.1097/MD.0000000000032685

**Published:** 2023-01-20

**Authors:** Emir Kaan İzci, Fatih Keskin

**Affiliations:** a Neurosurgery Department, Konya City Hospital, Konya, Turkey; b Neurosurgery Department, Necmettin Erbakan University Meram Faculty of Medicine, Konya, Turkey.

**Keywords:** coccydynia, coccyx fracture, coccyx trauma, readability

## Abstract

The aim of this research is to evaluate the websites containing “coccydynia, coccyx trauma or fracture” in terms of readability, reliability, accuracy, and quality. Searches for “coccydynia, coccyx trauma, coccyx fracture” were carried out in the 3 most used search engines in the USA: Google, Yahoo, and Bing in February 2022. A total of 141 websites were rated by 2 different neurosurgeons for the “Global Quality Score” and “Alexa Popularity Rank.” 97.2% of the sites examined include the definition of the disease, 66% include the importance of the disease, 92.9% include the symptoms of the disease, 88.7% include the treatment of the disease, 78% include the signs of the disease, 77.3% include the mechanism of the disease It has been determined that the websites examined within the scope of the research have high global quality score (GQS) and APR and are enriched with images to a large extent.

## 1. Introduction

The issue of determinants of the health status of individuals and societies is a topic that is frequently mentioned in the literature. According to general acceptance, the 4 main factors that affect health status are genetic factors, environmental factors, health services, and individuals’ behaviors. Blum expressed these factors as a model and added natural resources, population, cultural system, mental health, and ecological balance surrounding these factors.^[1,2]^ In addition, there are also factors that are expressed as social determinants of health status. These are expressed as non-medical factors that affect the health status of people, for example, the environment in which people are born, live, age, and work.^[3]^ Here, it is possible to evaluate each factor separately, and each of them has a significant impact on the health status. However, behaviors are among the factors that individuals determine themselves and can be changed. In this framework, the protection and improvement of the current health status and the prevention of health risks are closely related to behaviors. In this context, there are some risky behaviors as well as behaviors such as physical activity, sleep, and nutrition.^[4]^ One of the other important issues related to behavior is health-seeking behavior. It is expressed as health-seeking behavior or health information-seeking behavior. These behaviors are related to what kind of action individuals follow regarding their current situation when they perceive a negative situation regarding their health and from which sources they try to obtain information.^[5]^ When such a need arises, individuals can apply to professional health services as well as to traditional healers, relatives, or family elders.^[6]^ Today, it is known that the internet is used extensively as a source of information. With the developing computer technologies and the widespread use of the internet, the use of the internet has started to take place in almost every aspect of human life. Individuals can use the internet within the framework of their working life, for shopping purposes, to make payments, to communicate with other individuals, to receive education or training, to attend various meetings, or for entertainment. The rate of households with internet access has increased rapidly over the years.^[7–9]^ At the same time, it has become possible to conduct research on various subjects by using search engines. In this sense, it has become possible to access information on a wide variety of subjects through search engines. Individuals can conduct research on the internet about the health status of themselves or a relative.^[10]^ As a result of this research, they can access thousands of websites and many different kinds of information on the internet. Some of this information is available from in reliable sources such as public health authorities’ websites or the websites of private, foundation, or public health institutions involved in the delivery of official health services. Other sources, on the other hand, are sources whose reliability is uncertain. It is known that such sources also contain information written by individuals who do not have any professional knowledge, based on their personal comments or beliefs. For this reason, the behavior of searching for health information on the internet requires a high degree of health literacy, as well as reliable and quality content.^[11–13]^ Subjects such as “coccydynia, coccyx trauma, coccyx fracture,” which are the subject of this research, are among the subjects that individuals want to get information about. The coccyx is a triangular bone formed by the fusion of 3 to 5 segments of bone and is the most extreme segment of the spine. It got its name from the “coccyx” because it resembles the beak of the cuckoo, which is called “cuckoo” in Greek. It usually consists of 4 segments, rarely 5 segments. These segments are mobile at birth. Distal segments fuse in childhood and proximal segments in early adulthood.^[14]^ Coccydynia means pain in the coccyx. The cause of pain in this bone is usually trauma. Particularly, pain occurs when this bone is fractured or dislocated as a result of falling in a sitting position or when the coccyx becomes mobile by force during birth delivery. It mostly manifests itself as severe pain felt while sitting, and sometimes as a pain that increases with standing, and it reduces the quality of life of individuals.^[15,16]^ Individuals experiencing this problem can perform online searches related to the related problem. As a result of these searches, they have access to information on a number of websites. Although there are many publications on readability in the literature, it was seen that there was no such a study on coccyx and coccyx traumas. In addition, although it is frequently seen in the society, there is a lot of wrong information about the treatment management. In this context, the aim of this research is to evaluate the website contents accessed by scanning the common ailments such as “coccydynia, coccyx trauma, coccyx fracture” in search engines in terms of readability, reliability, accuracy, and quality.

## 2. Methods

On February 26, 2022, a search for common disorders such as “coccydynia, coccyx trauma, coccyx fracture” was carried out on Google, Yahoo, and Bing search engines, which are the 3 most used search engines in the United States (USA). As a result of the scans made in these search engines, websites containing information about patients and containing analyzable text were included in the study. Those that are prepared for health professionals contain only videos, being in the form of newspaper news, whose content language is not English, those that are limited to subscription, containing conference papers, and containing academic education are excluded. A total of 141 websites were included in the research. Included websites were rated by 2 different neurosurgeons for the “Global Quality Score.” In undecided cases, the opinion of a third neurosurgeon was consulted and the final decision was made. The popularity of the websites was evaluated and classified according to the number of clicks by scanning them on the “Alexa Popularity Rank” site. Besides, the websites included in the research were classified according to whether they had the “Health on the Net Foundation” code or not. Websites were also evaluated for Flesch Reading Ease (FRES), Gunning FOG, Flesch-Kincaid Grade Level (FKGL), Coleman-Liau Index (CLI), Simple Measure of Gobbledygook (SMOG), automated readability index (ARI), and Linsear Write Formula (LWF) scores. Then, it was determined whether there was information such as the definition of the disease, the importance of the disease, symptoms of the disease, treatment methods of the disease, findings, and occurrence of the disease and mechanism of the disease on the website.

### 2.1. Statistical analyses

The data obtained within the scope of the research were analyzed by statistical package for social sciences 22.0 (IBM Inc. Chicago, IL) software. The descriptive statistics were calculated and presented as the median (interquartile range) and the frequency (percentage). The normality analysis was carried out with the Kolmogorov-Smirnov test, and the numerical data were not suitable for normal distribution (*P* < .05). Therefore, comparisons between groups were calculated with the Mann–Whitney *U* test. Chi-Square with Yates correction and Fisher’s Exact Test were used to analyze the relationships between categorical variables. *P* < .05 values were considered statistically significant results.

### 2.2. Ethical approval

Since the study was about internet searches, ethical approval has not been taken. In these types of searches, the data are obtained from the search results. Therefore, no need to apply for ethical approval.

## 3. Results

The median global quality score (GQS) for the examined websites is 5 (IQR:3–6) and the median APR is 297766 (IQR:7707–1444252). 53.9% of them have APR above 250000, 29.8% below 25000, and 16.3% between 25000 and 250000. The content of 77.3% of the sites examined was produced by health professionals and 24.1% of them have HON codes. 46.8% of these sites provided references as content sources, and 70.9% were enriched with illustrations and pictures (Table 1).

**Table 1:** Parameters assessing the quality of sites included in the study

QUALITY (n)		141
Global quality score (median, IQR)		5 (3–6)
Alexa popularity rank (median, IQR)		297766 (7707–1444252)
APR groups (n, %)	<25000	42 (29.8)
	25000–250000	23 (16.3)
	>250000	76 (53.9)
Characteristics by the source of upload (n, %)	Health professional	109 (%77.3)
	Non-medical professional	32 (%22.7)
HON code (n, %)	Yes	34 (%24.1)
	No	107 (%75.9)
Originality (n, %)	Referencing	66 (%46.8)
	No referencing at all	75 (53.2)
Illustration and pictures (n, %)	Yes	100 (%70.9)
	No	41 (%29.1)

APR = Alexa popularity rank, IQR = interquartile range (25%–75%).

While the readability scores do not differ according to the characteristics of the source of upload, there are statistically significant differences in terms of the quality scores of GQS, APR, HON Code, and originality (*P* < .01). Sites whose content is produced by healthcare professionals have a higher GQS score and a lower APR score. Similarly, HON Code, originality, illustrations, and pictures are higher on these websites.

According to Characteristics by the source of upload, the importance of the disease (*P* < .01), the symptoms of the disease (*P* < .05), the treatment of the disease (*P* < .01), the signs of the disease (*P* < .01), and the mechanism of the disease (*P* < .01) were found to be statistically significantly higher (Table 2).

**Table 2:** The distribution of the parameters of readability, quality and adequacy of the information on the sites included in the research according to the upload status by the health professional

	Characteristics by the source of upload		*P*
	Health professional (n = 109)	Non-medical professional (n = 32)	
**Readability,** median (IQR)			
FRES	43.1 (32.6–53.1)	46.8 (36.2–58.3)	.089†
Gunning FOG	13.6 (11.4–15.6)	12.6 (11.2–16.9)	.933†
FKGL	12.2 (10–14.9)	11.5 (8.9–13.7)	.176†
CLI	12 (11–13)	12 (11–12)	.400†
SMOG	10.3 (9.1–11.6)	9.8 (8.4–11.9)	.459†
ARI	12.2 (9.3–14.7)	11.3 (9.4–14.1)	.570†
LWF	12.8 (9.1–15.7)	12 (8.7–17.1)	.654†
**Quality**			
GQS, median (IQR)	5 (5–6)	3 (2–4)	<.001†^,^**
APR, median (IQR)	67020 (5825–914691)	1324564 (146886–3180771)	.001†^,^**
HON code, n (%)	34 (%31.2)	0 (%0)	.001‡^,^**
Originality, n (%)	62 (%56.9)	4 (%12.5)	.000‡^,^**
Illustration and pictures, n (%)	78 (%71.6)	22 (%68.8)	.931‡
**Content sufficiency,** n (%)			
Description of the disease	107 (%98.2)	30 (%93.8)	.222§
Importance of the disease	90 (%82.6)	3 (%9.4)	<.001‡^,^**
Symptoms of the disease	104 (%95.4)	27 (%84.4)	.048§^,^*
Treatment of the disease	102 (%93.6)	23 (%71.9)	.002§^,^**
Signs of the disease	97 (%89)	13 (%40.6)	<.001‡^,^**
The mechanism of the disease	95 (%87.2)	14 (%43.8)	<.001‡^,^**

% = column percentage, APR = Alexa popularity rank, ARI = automated readability index, CLI = Coleman-Liau Index, FKGL = Flesch-Kincaid Grade Level, FRES = Flesch reading ease, GQS = global quality score, Gunning FOG = Gunning Fog Index, LWF = Linsear Write Formula, SMOG = simple measure of gobbledygook.

† Mann–Whitney *U* test.

‡ Yates’ Chi-Square.

§ Fishers’ exact test.

* *P* < .05.

** *P* < .01.

There are statistically significant differences in terms of FRES (*P* < .05), Gunning FOG (*P* < .01), FKGL (*P* < .01), SMOG (*P* < .01), ARI (*P* < 0, 01), and LWF (*P* < .01) depending on whether the sites have HON Code or not. There is no statistically significant difference in terms of CLI (*P* > 0,05). Sites with HON Code have higher FRES and Gunning FOG, while FKGL, SMOG, ARI, and LWF are lower. There are statistically significant differences in terms of the quality scores of GQS, APR, characteristics by the source of upload, originality (*P* < .01), and inclusion of illustrations and pictures (*P* < 0,05). Sites with HON Code have higher GQS while APR, uploading by health professionals, originality, and inclusion of illustrations and pictures are lower.

Among the content sufficiency criteria, the importance of the disease (*P* < 0,01), the treatment of the disease (*P* < 0,05), the signs of the disease (*P* < 0,01), and the mechanism of the disease (*P* < 0,01) have a statistically significant difference according to whether or not they have the HON code (*P* < .01), while there is no statistically significant difference in terms of the definition and symptoms of the disease (*P* > 0,05). The importance, treatment, signs, and mechanism of the disease are lower in those with HON Codes (Table 3).

**Table 3:** The distribution of the parameters of readability, quality and adequacy of the sites included in the research according to the quality accreditation status

	HON code		*P*
	Yes (n = 34)	No (n = 107)	
**Readability,** median (IQR)			
FRES	58.225 (46.3–73.45)	42.7 (34.1–52.5)	.038†^,^*
Gunning FOG	14.25 (11.4–16.8)	13.8 (11.8–16.2)	.001†^,^**
FKGL	10.5 (8.9–12.825)	12.6 (10.2–14.9)	.001†^,^**
CLI	11.5 (10–12)	12 (11–13)	.314†
SMOG	9.3 (8075–10.8)	10.8 (9.2–12)	.002†^,^**
ARI	9.55 (8.1–12.45)	12.7 (9.9–14.9	<.001†^,^**
LWF	9.2 (7675–13.8)	13.4 (9.4–17.1)	<.001†^,^**
**Quality**			
GQS, median (IQR)	6 (5.75–6)	4 (3–5)	<.001†^,^**
APR, median (IQR)	6417.5 (1981.25–22545)	776813 (142915–2590927)	<.001†^,^**
HON Code, n (%)	34 (31.2)	75 (%68.8)	.001‡^,^**
Originality, n (%)	29 (%43.9)	37 (%56.1)	<.001‡^,^**
Illustration and pictures, n (%)	30 (%30)	70 (%70)	.020‡^,^*
**Content Sufficiency,** n (%)			
Description of the disease	34 (%24.8)	103 (%75.2)	.572§
Importance of the disease	33 (%35.5)	60 (%64.5)	<.001‡^,^**
Symptoms of the disease	34 (%26)	97 (%74)	.118§
Treatment of the disease	34 (%27.2)	91 (%72.8)	.012§^,^*
Signs of the disease	33 (%30)	77 (%70)	.005‡^,^**
The mechanism of the disease	33 (%30.3)	76 (%69.7)	.003‡^,^**

% = column percentage, APR = Alexa popularity rank, ARI = automated readability index, CLI = Coleman-Liau Index, FKGL = Flesch-Kincaid Grade Level, FRES = Flesch reading ease, GQS = global quality score, Gunning FOG = Gunning Fog Index, IQR = interquartile range (25%–75%), LWF = Linsear Write Formula, n = site number, SMOG = simple measure of gobbledygook.

† Mann–Whitney *U* test.

‡ Yates’ Chi-Square.

§ Fishers’ exact test.

* *P* < .05.

** *P* < .01

The FRES median for the examined websites is 43,8 (IOR: 35.15–54.2), Gunning FOG median is 13,5 (IOR: 11.3–15.9), FKGL median is 12.1 (IOR: 9.7–14.1), CLI median is 12 (IOR: 10–13), SMOG median is 10.1 (IOR: 8.6–11.8), ARI median is 11.8 (IOR: 9.4–14.4), and LWF median is 12.8 (IOR: 8.95–15.8). 97.2% of the sites examined include the definition of the disease, 66% include the importance of the disease, 92.9% include the symptoms of the disease, 88.7% include the treatment of the disease, 78% include the signs of the disease, 77.3% include the mechanism of the disease. Statistically significant relationships were found between whether the websites provide references and whether they include the importance of the disease, the treatment of the disease, the signs of the disease, the mechanism of the disease, and illustrations and pictures (*P* < .01). The use of illustrations and pictures, providing the importance of the disease, the treatment of the disease, the signs of the disease, and the mechanism of the disease are higher in websites with references. On the other hand, there was no statistically significant relationship in terms of the definition of the disease and the symptoms of the disease (*P* > .05). FRES, Gunning FOG, FKGL, CLI, SMOG, ARI, and LWF scores do not have a statistically significant difference according to providing references (*P* > .05). Statistically significant relationships were found between the importance of the disease (*P* < .01), the treatment of the disease, the signs of the disease, the mechanism of the disease, and the use of illustrations and pictures (*P* < .05). The importance of the disease, the treatment of the disease, the signs of the disease, and the mechanism of the disease are higher on websites that use illustrations and pictures. On the other hand, FRES, Gunning FOG, FKGL, CLI, SMOG, ARI, and LWF scores do not show a statistically significant difference according to the use of illustration and pictures (*P* > .05). FRES, Gunning FOG, FKGL, CLI, SMOG, ARI, and LWF scores do not show a statistically significant difference according to APR groups (*P* > .05). Statistically significant relationships were found between APR Groups and inclusion of the importance of the disease (*P* < .01), the treatment of the disease, the signs of the disease, the mechanism of the disease, HON code, illustration, and pictures, and references (*P* < .01). On the other hand, there was no statistically significant relationship between APR groups and the definition and symptoms of the disease (*P* > .05). As the APR interval increases, the level of inclusion of the disease’s importance, treatment, signs, mechanism, references, and HON code decreases. The level of including illustration and pictures is lower in 25000 to 250000 and 250000 and above groups compared to the 0 to 25000 group.

## 4. Discussion

When individuals experience some health problems, they can seek information to eliminate uncertainties regarding these problems and find solutions. This information seeking behavior usually takes place online today. In parallel with the widespread use of the internet from the past to the present, the use of the internet as a source of health information has also become widespread.^[17]^ For this reason, the health information on the websites should be seen as frequently referenced information sources and should provide high-quality, readable, and reliable information in this context. Because of the widespread use of the internet, the reliability of health information on the internet has become questionable.^[18]^

Daraz et al concluded in their study that online health information is not suitable for general use in the United States and Canada.^[19]^ They state that poor readability can lead to misinformation and poor health outcomes. Today, individuals are exposed to many different variables in many different areas. Obtaining information from many different sources, and interpreting and evaluating this information has become a necessity. For this reason, it is necessary to have different kinds of literacy in the evaluation of the information obtained in many different fields.^[20]^ Health literacy is one of the important issues in terms of protecting and improving the health status of individuals, as well as regaining deteriorated health.^[21]^ Individuals with low health literacy may have difficulties in finding, understanding, and interpreting health information, as well as applying it. Therefore, health-related websites should present appropriate information with high readability and reliability. Because even an individual with a high level of education and good general literacy may experience problems in reading and understanding health-related information.

Otu and Karagözoğlu (2022), in their research, found that the content of Turkish websites that provide information to patients about fibromyalgia syndrome is weak and of low quality.^[22]^ It is also stated to have low readability. Similarly, Deniz et al (2020) found that the readability level of the information texts about the triple test on the websites was low.^[23]^ Akbulut (2022), on the other hand, reached the conclusion that the readability of the texts on transparent plaques on the internet is of medium difficulty.^[24]^ In another study, it was determined that the readability level of skin cancer patient information texts on websites was of medium difficulty and insufficient in terms of content. It has been stated that the revision of these websites, taking into account the health literacy level of the general population, may contribute to early cancer diagnosis.^[25]^ It has been concluded that the websites examined within the scope of the current research have a high GQS score in general. It has been determined that 53% of the sites are high in terms of APR (250.000 and above), and the content of 77.3% is prepared by health professionals. Although only 24.1% of the websites have HON codes, 46.8% have references and in more than 70% of the websites information was enriched by illustrations. It was determined that 92% of the websites included the definition of the disease, 66% importance of the disease, 92.9% symptoms of the disease, 88.7% treatment of the disease, 78% signs of the disease, and 77.3% mechanism of the disease. Therefore, it can be stated that the websites included in the research generally have a high-quality score and the number of clicks is high as well. At the same time, it is a positive situation that most of the site content is prepared by health professionals. In addition to the useful health information on the internet, there is also intense information pollution. The rate of websites with HON codes is low. According to the research findings, it can be recommended to include HON code on websites because of the higher readability rate of sites with HON code and higher GQS. However, since the sites with the HON code are lagging behind in terms of content adequacy, it can be stated that the content adequacy of the sites containing the HON code should be increased. When evaluated in terms of content, it has been determined that most of the websites include information such as the definition, importance, symptoms, treatment, and mechanism of diseases. Although this indicates that the content adequacy is high, it is clear that the content quality should also be supported in this sense.

People who provided the content of the websites are among the important findings obtained in the research. Accordingly, content created by healthcare professionals has a higher GQS score. It has also more originality and has more of the HON code. Moreover, the importance of the disease, symptoms of the disease, treatment methods, and mechanism of the disease are more likely to be included in the websites whose content is created by health professionals. For websites to be of high quality, readability and reliability, it can be recommended that the contents be created by health professionals. McInnes and Haglund (2011) found that websites with gov extensions have high readability, while websites with “.edu” extensions have low readability.^[26]^ Similarly, it was stated here that high-quality and plain language websites should be created by health professionals.

The use of illustrations and pictures that explain the importance of the disease, the treatment of the disease, the symptoms of the disease, and the mechanism of the disease is more on the websites with references. When individuals experience health problems, they want to have information about their diseases. Today, with the widespread use of technology, online platforms are frequently used because of the fast and easy access to information. Therefore, it can be suggested that in order to increase the quality of websites, they should include references and at the same time increase the use of images and illustrations. As a result, it has been determined that the websites examined within the scope of the research have high GQS and APR, and are enriched with images and illustrations to a large extent, but the content quality is insufficient. For this reason, it can be suggested that the content quality of the relevant websites should be increased and they should be arranged in a safe and convenient manner for the general public.

## Author contributions

**Conceptualization:** Emir Kaan İzci.

**Formal analysis:** Fatih Keskin.

**Methodology:** Emir Kaan İzci.

**Software:** Fatih Keskin.

**Validation:** Fatih Keskin.

**Writing – original draft:** Emir Kaan İzci.

**Writing – review & editing:** Emir Kaan İzci, Fatih Keskin.
